# Association between fatigue and motor progression in Parkinson’s disease in southern Chinese

**DOI:** 10.1007/s10072-019-04059-z

**Published:** 2019-09-04

**Authors:** Hong-Xiang Yu, Meng-Ruo Guo, Gang Li, Bei Zhang

**Affiliations:** Department of Neurology, East Hospital, Tongji University School of Medicine, 1800 Yuntai Road, Shanghai, 200123 China

**Keywords:** Parkinson’s disease, Motor function, Autonomic dysfunction, Disease progression

## Abstract

**Study objectives:**

The aim was to investigate whether fatigue could predict the development of motor symptoms of Parkinson’s disease (PD) in a southern Chinese population.

**Methods:**

In total, 246 PD patients were recruited. All patients were evaluated by Fatigue Severity Scale (FSS), Hamilton Anxiety Rating Scale, Hamilton Depression Rating Scale, and Unified PD Rating Scale provided by Movement Disorders Society (MDS-UPDRS). MDS-UPDRS was re-evaluated after 2 years.

**Results:**

FSS scores were associated with total score and subparts of MDS-UPDRS (total: *p* 0.039, *p* 0.030, adjusted; part III: *p* 0.022, *p* 0.016, adjusted).

**Conclusions:**

The symptom of subjective fatigue could predict the progression of PD.

## Introduction

Parkinson’s disease (PD) is a common movement disorder characterized by bradykinesia, rigidity, and resting tremors [1]. PD symptoms are divided into two parts: motor symptoms, non-motor symptoms [2]. Non-motor symptoms vary from people to people, and there are several different non-motor symptoms in PD, such as autonomic symptoms and fatigue.

Fatigue is a common disabling symptom but easily ignored in PD [3, 4]. Half of all PD patients were influenced by fatigue [5]. Subjective fatigue is divided into physical fatigue and mental fatigue [6]. Fatigue could be the first symptom in PD patients. However, the relationship between fatigue and disease progression has not been discovered. Besides, biomarkers predicting the progression of PD are unclear. It is beneficial to be aware of the progression of PD, which is useful for management and nursing.

In this study, we investigated the association between fatigue and motor progression in PD.

## Methods

### Study population

PD patients were collected from the outpatient clinic of Shanghai East Hospital and were diagnosed by movement disorder specialists based on diagnostic criteria brought up by Movement Disorders Society (MDS) [7]. We also recorded Hoehn–Yahr staging and their disease duration. Patients with Hoehn–Yahr staging between 1.0 and 2.0 were regarded as having mild PD. Several common secondary causes of parkinsonism were excluded, such as inflammatory, drug-induced, vascular, and toxin-induced parkinsonism. Parkinsonism with other neurodegenerative diseases, such as progressive supranuclear palsy (PSP), cerebral-basal degeneration (CBD), and multiple system atrophy (MSA), was also excluded. We also excluded patients with anxiety or depression using Hamilton Anxiety Rating Scale (HARS) and Hamilton Depression Rating Scale (HDRS). All participants signed consent forms. This study was approved by the ethics committee of Shanghai East Hospital.

### Evaluation

Every PD patient included in this study received evaluation including the following rating scales: Unified PD Rating Scale provided by Movement Disorders Society (MDS-UPDRS) was used to assess the status of PD [8]. Fatigue Severity Scale (FSS) was used to assess fatigue. Hamilton Anxiety Rating Scale and Hamilton Depression Rating Scale were used to assess anxiety and depression, respectively. Researchers received strict training on these scales before assessing PD patients. We re-evaluated them with MDS-UPDRS door to door after 2 years of baseline for assessing the status of PD. Motor fluctuations and dyskinesia were also recorded at the baseline and the follow-up.

### Statistics

R (version 3.5.0), stats package (version 3.5.0), was used to perform statistical analysis. Number and percentage were used to describe categorical variables. Mean and standard deviation (SD) were used to describe numerical variables and described in the form mean ± SD. Univariate regression analysis and multivariate regression analysis were performed to investigate the association between scores of autonomic function and changes in MDS-UPDRS (total scores and subparts) between baseline and follow-up. We adjusted age, gender, disease duration, and Hoehn–Yahr staging in the multivariate regression analysis.

## Results

There were 246 PD patients who participated in this study. Their average age was 57.07 ± 10.01 years. A total of 103 (41.87%) patients were female. Disease duration of PD patients included in this study was 4.82 ± 4.19 years. Thirty-seven (15.04%) of them reported their family history of PD. A total of 180 (73.17%) of them had mild PD. Seven (2.85%) patients had dyskinesia and 27 (10.98%) patients had motor fluctuations at the baseline. After 2 years, there were still 7 (2.85%) patients who had dyskinesia. Five more patients had motor fluctuations. Their FSS scores were 30.63 ± 18.32. There was progression of MDS-UPDRS total scores and their subparts except part IV (total score 46.86 ± 25.52, follow-up 55.38 ± 28.05; part I 7.02 ± 5.85, follow-up 8.15 ± 6.16; part II 11.92 ± 8.02, follow-up 14.78 ± 8.83; part III 27.90 ± 16.48, follow-up 32.43 ± 17.61) (Table 1).

**Table 1:** Demographic data presented in this study

	PD patients included in this study (*N* = 246)
Age, mean (SD)	57.07 (10.01)
Gender, female, *N* (%)	103 (41.87)
Disease duration at the baseline, mean (SD)	4.82 (4.19)
Family history, *N* (%)	37 (15.04)
Hoehn–Yahr Staging, *N* (%)	
1.0	55 (22.36)
1.5	38 (15.45)
2.0	87 (35.37)
2.5	42 (17.07)
3.0	18 (7.32)
4.0	6 (2.44)
5.0	0 (0)
Dyskinesia, *N* (%)	7 (2.85)
Motor fluctuations, *N* (%)	27 (10.98)
Dyskinesia, *N* (%)*	7 (2.85)
Motor fluctuations, *N* (%)*	33 (13.41)
HARS, mean (SD)	1.48 (1.13)
HDRS, mean (SD)	1.43 (1.19)
FSS, mean (SD)	30.63 (18.32)
MDS-UPDRS, mean (SD)	46.86 (25.52)
Part I	7.02 (5.85)
Part II	11.92 (8.02)
Part III	27.90 (16.48)
Part IV	0.02 (0.14)
MDS-UPDRS, mean (SD)*	55.38 (28.05)
Part I	8.15 (6.16)
Part II	14.78 (8.83)
Part III	32.43 (17.61)
Part IV	0.02 (0.14)

*FSS*, Fatigue Severity Scale; *HARS*, Hamilton Anxiety Rating Scale; *HDRS*, Hamilton Depression Rating Scale; *MDS*, Movement Disorders Society; *PD*, Parkinson’s disease; *SD*, standard deviation; *UPDRS*, Unified Parkinson’s Disease Rating Scale

*The follow-up status

With linear regression, the association between FSS at the baseline and the progression of MDS-UPDRS was calculated. FSS score was associated with MDS-UPDRS total score and part III no matter if with or without adjustment (FSS vs. MDS-UPDRS total score: *p* value 0.039, coefficient 0.041, without adjustment; *p* value 0.030, coefficient 0.036, with adjustment; FSS vs. MDS-UPDRS part III: *p* value 0.022, coefficient 0.028, without adjustment; *p* value 0.016, coefficient 0.025, with adjustment). However, there were no statistically significant results when we assessed the association between FSS and MDS-UPDRS part I/II (Table 2).

**Table 2:** Association between FSS at the baseline and the difference of MDS-UPDRS between the follow-up and baseline

	FSS score			
	*p* value	Coefficient	*p* value*	Coefficient*
MDS-UPDRS total score	0.039	0.041	0.030	0.036
MDS-UPDRS part I	0.807	0.001	0.808	0.001
MDS-UPDRS part II	0.095	0.011	0.080	0.010
MDS-UPDRS part III	0.022	0.028	0.016	0.025
MDS-UPDRS part IV	–	–	–	–

*FSS*, Fatigue Severity Scale; *MDS*, Movement Disorders Society; *UPDRS*, Unified Parkinson’s Disease Rating Scale

*Adjusted for age, gender, duration, and Hoehn–Yahr staging

## Discussion

Our study found that subjective fatigue severity score was associated with the progression of motor function of Parkinson’s disease. To our knowledge, this was the first study focusing on the association between fatigue and the progression of motor function.

Fatigue in PD is associated with multiple neurotransmitter pathways. Dopaminergic treatment is effective for fatigue in PD [9, 10]. Striatal ^11^C-dihydrotetrabenazine (DTBZ) uptake was a significant predictor of fatigue in mild PD, which indicated the involvement of monoaminergic pathways [11]. In the serum of PD patients, the levels of 5-hydroxytryptamine (5-HT) and transferrin are markedly decreased in the fatigue group [12].

Fatigue in PD patients influenced more regions than in PD patients without fatigue. Brain areas including frontal, temporal, and parietal regions indicative of emotion, motor, and cognitive functions are involved in fatigue in PD patients (hypermetabolism in the right middle temporal gyrus and left middle occipital gyrus; hypometabolism in the right precuneus, left inferior frontal gyrus, and left superior frontal gyrus) [13, 14]. In depressed PD patients, the prevalence of fatigue was higher than in non-depressed PD patients [15]. Dopaminergic pathway is involved in depression. Besides, the presence of fatigue was associated with the presence of autonomic dysfunction [16, 17]. Moreover, the level of α-synuclein oligomer was higher and that of Aβ_1-42_ was lower in the fatigue group in the cerebrospinal fluid [18].

Our study found that higher involvement of subjective fatigue severity score could predict motor functions in PD. Higher involvement of subjective severity could reflect wider spread of α-synuclein, which could influence faster progression of motor function.

The strengths of our study are that motor functions of PD were assessed by a structured scale which is widely accepted. The diagnosis was based on MDS criteria. We performed a 2-year follow-up because the progression could be easily observed.

This study has some weakness and limitations. First, we did not take other structured questionnaires such as The Multidimensional Fatigue Inventory. Questions of the Multidimensional Fatigue Inventory and questions of the FSS were not identical. Second, we did not take objective methods to evaluate PD motor function. Third, the sample of our study is small and our study was a single-center study. More multicenter and larger studies are warranted.

Ideally, fatigue should be measured early near the onset of disease to better evaluate motor progression, and follow-up should be longer. However, it is very hard to operate for now. Many patients are not seeking a doctor when they suffer fatigue in China. Even though their motor symptoms are present, they choose to stay at home and sleep rather than seeing a doctor. More multicenter and larger studies on this design are warranted.

In conclusion, fatigue could predict the progression of PD. Larger multicenter studies are warranted.
